# Proteomics Analysis to Identify and Characterize the Molecular Signatures of Hepatic Steatosis in Ovariectomized Rats as a Model of Postmenopausal Status

**DOI:** 10.3390/nu7105434

**Published:** 2015-10-22

**Authors:** Chen-Chung Liao, Yen-Shuo Chiu, Wan-Chun Chiu, Yu-Tang Tung, Hsiao-Li Chuang, Jyh-Horng Wu, Chi-Chang Huang

**Affiliations:** 1 Proteomics Research Center, National Yang-Ming University, Taipei 11221, Taiwan; ccliao@ym.edu.tw; 2 Graduate Institute of Sports Science, National Taiwan Sport University, Taoyuan 33301, Taiwan; 1021301@ntsu.edu.tw (Y.-S.C.); peggytung@ntsu.edu.tw (Y.-T.T.); 3 Department of Orthopedic Surgery, Taipei Medical University Shuang Ho Hospital, New Taipei City 23561, Taiwan; 4 School of Nutrition and Health Sciences, Taipei Medical University, Taipei 11031, Taiwan; wanchun@tmu.edu.tw; 5 National Laboratory Animal Center, National Applied Research Laboratories, Taipei 11529, Taiwan; p650214@nlac.narl.org.tw; 6 Department of Forestry, National Chung Hsing University, Taichung 40227, Taiwan

**Keywords:** liver, proteome, ingenuity pathways analysis

## Abstract

Postmenopausal women are particularly at increased risk of developing non-alcoholic fatty liver disease (NAFLD). Here we aimed to determine the impact of postmenopausal-induced NAFLD (PM-NAFLD) in an ovariectomized rat model. Sixteen six-week-old Sprague-Dawley female rats were randomly divided into two groups (eight per group), for sham-operation (Sham) or bilateral ovariectomy (Ovx). Four months after surgery, indices of liver damage and liver histomorphometry were measured. Both serum aspartate aminotransferase (AST) and alanine aminotranferease (ALT) levels were significantly higher in the Ovx than Sham group. We performed quantitative LC-MS/MS-based proteomic profiling of livers from rats with PM-NAFLD to provide baseline knowledge of the PM-NAFLD proteome and to investigate proteins involved in PM-NAFLD by ingenuity pathways analysis (IPA) to provide corroborative evidence for differential regulation of molecular and cellular functions affecting metabolic processes. Of the 586 identified proteins, the levels of 59 (10.0%) and 48 (8.2%) were significantly higher and lower, respectively, in the Ovx group compared to the Sham group. In conclusion, the changes in regulation of proteins implicated in PM-NAFLD may affect other vital biological processes in the body apart from causing postmenopause-mediated liver dysfunction. Our quantitative proteomics analysis may also suggest potential biomarkers and further clinical applications for PM-NAFLD.

## 1. Introduction

Nonalcoholic fatty liver disease (NAFLD) is characterized by the focal or diffuse accumulation of fat in the liver parenchyma of patients who deny abusive alcohol consumption [1]. The prevalence of NAFLD increases with age, obesity, and postmenopausal status [2]. Premenopausal women have lower risk of cardiovascular disease as compared with BMI-matched men. The lower risk may be due to estrogen’s ability to limit liver fat accumulation and stimulate fat oxidation, thereby preventing hepatic insulin resistance [3,4,5,6,7,8,9]. Recent studies showed that total loss of estrogen signaling increases liver fat and liver lipid infiltration in humans with estrogen receptor α (ERα) mutations, Ovx (ovariectomized) rodents, mice with global ERα knockout, and mice lacking aromatase [10,11,12,13,14]. Mittendorfer *et al*., showed that the estrogen deficiency causes body fat redistribution, with the accumulation of visceral fat, which can affect the development and progression of NAFLD [5].

Because the liver plays a crucial role in metabolism, a comparative proteomic analysis of the hepatic response in Ovx rat model can help illustrate postmenopausal-induced NAFLD (PM-NAFLD). Proteomics is a large-scale comprehensive study of proteins, including information on protein abundance and modification along with their interacting networks [15]. It is a powerful tool for studying changes in protein expression and identifying biomarkers for pathogenic processes [16,17,18]. To our knowledge, no precise mechanism has been identified to explain NAFLD in post-menopausal women [19].

The aim of this study was to explore the impact of PM-NAFLD in an Ovx rat model by shotgun proteomic analysis. We hoped to identify a set of differentially expressed proteins as molecular markers for PM-NAFLD.

## 2. Experimental Section

### 2.1. Animals and Experiment Design

Specific pathogen-free female Sprague Dawley (SD) rats (eight weeks old) were purchased from BioLASCO (A Charles River Licensee Corp., Yi-Lan, Taiwan). All animals were given a standard AIN-93 purified diet as described [20] with some modification, distilled water *ad libitum*, housed at room temperature (23 ± 2 °C) with humidity control (55% ± 10%) with a 12-h light/12-h dark cycle. The modified AIN-93 diet contained 140 g casein, 100 g corn oil, 405.7 g corn starch, 155 g dextrin, 100 g sugar, 35 g mineral mixture, 10 g vitamin mixture, 50 g methyl cellulose, 2.5 g choline bitrate, and 1.8 g l-Cystine/kg diet. After acclimation for one week, the rats were anesthetized by intraperitoneal injection of Zoletil/Xylazine (30 mg/kg Zoletil with 10 mg/kg Xylazine). The bilateral ovariectomization was according to a standard CAF procedure. Accordingly, the other eight rats underwent sham operation and were designated the Sham group. Animals were anesthetized with Zoletil/Xylazine and killed four months after ovariectomy. Blood samples were collected from the abdominal aorta, and the liver tissues were carefully harvested, rinsed in ice-cold normal saline, blotted dry and stored at −80 °C for further analysis. All animal experiments adhered to the guidelines of the Institutional Animal Care and Use Committee (IACUC) of Taipei Medical University (TMU). The IACUC ethics committee approved this study under the protocol IACUC-LAC-99-251.

### 2.2. Determination of Blood Biochemical Variables

We evaluated the effect of Ovx on plasma estradiol, total cholesterol (TC), triacylglycerol (TG), aspartate aminotransferase (AST) and alanine aminotranferease (ALT) levels at four months after ovariectomy. The plasma was prepared by centrifugation at 1500× *g*, 4 °C for 15 min. Plasma TC, TG, AST, and ALT levels were determining by use of an auto-analyzer (Hitachi 7060, Hitachi, Japan). Plasma concentration of estradiol was analyzed by using a radioimmunoassay kit (Diagnostic Products Corporation, Los Angeles, CA, USA).

### 2.3. Gross and Histological Evaluation of Liver Tissues

Livers were fixed in 10% formalin, embedded in paraffin and cut into 4-ìm thick slices as per our previous study [21]. Tissue sections were stained with hematoxylin and eosin (H&E) or Masson trichrome and examined using a light microscope equipped with a CCD camera (Olympus BX51; Olympus Co., Ltd., Tokyo, Japan).

### 2.4. Protein Sample Preparation

Each rat liver samples (100 mg) was placed in a 2 mL sample tube contain ceramic beads (0.2 g, 1 mm diameter) and homogenized in cold 50 mM Tris buffer (pH 6.8) containging 1% SDS, 1× protease inhibtor (Complete, Roche, USA), and 2× PI2 (PhosSTOP, Roche, USA) with a Precellys^®^ 24 grinder (Bertin technologies, France). The tissue debris was removed by centrifugation at 15,000 rpm for 10 min at 4 °C, then transferred the supernatant to the new eppendorf. Protein concentration was measured by using BCA protein assay kit (Thermo, Rockford, IL, USA).

### 2.5. SDS-PAGE and In-Gel Digestion

The protein samples were resolved by 10% SDS-PAGE as previously described [22]. Briefly, a total of 50 μg of each protein sample was applied to the gel in triplicate, and the sizes of proteins were visualized by staining with Coomassie Brilliant Blue G-250 (Bio-Rad, Hercules, CA, USA). After electrophoresis. The gel lanes were split up into 10 equal fractions, and the slices were destained by repeatedly washing in a solution of 25 mM ammonium bicarbonate and 50% (V/V) acetonitrile (1:1) until the protein bands were invisible. After completely being dried with a Speed-Vac (Thermo Electron, Waltham, MA, USA), proteins in the gel fragments were then subjected to the reduction and cysteine alkylation reactions for irreversibly breaking disulfide bridges in the proteins. For the reduction, each gel piece was rehydrated with 2% (V/V) β-mercaptoethanol in 25 mM ammonium bicarbonate and incubated at room temperature for 20 min in the dark. Cysteine alkylation was performed by adding an equal volume of 10% (V/V) 4-vinylpyridine in 25 mM ammonium bicarbonate and 50% (V/V) acetonitrile for 20 min. The samples were than washed by soaking in 1 mL of 25 mM ammonium bicarbonate for 10 min. Following Speed-Vac drying for 20 min, in-gel trypsin digestion was carried out by incubating the samples with 100 ng of modified trypsin (Promega, Mannheim, Germany) in 25 mM ammonium bicarbonate at 37 °C overnight. The supernatant of the tryptic digest was transferred to an Eppendorf tube. Extraction of the remaining peptides from the gel involved adding 25 mM ammonium bicarbonate in 50% (V/V) acetonitrile, then collecting the solution after incubation for 10 min. The resulting digests were dried in a Speed-Vac and stored at −20 °C.

### 2.6. Nanoflow Ultra High-Performance Liquid Chromatography−Tandem Mass Spectrometry (nUPLC−MS/MS)

Each cryo-stored tryptic digest was resuspended in 30 μL of 0.1% (V/V) formic acid and analyzed using an online nanoAcquity ultra Performance LC (UPLC) system (Waters, Manchester, UK) coupled to a hybrid linear ion trap Orbitrap (LTQ-Orbitrap Discovery) mass spectrometer with a nanoelectrospray ionization source (Thermo Scientific, San Jose, CA, USA). After loading the sample with single injection model into the UPLC, the peptides were captured and desalted on a C18 trapping cartridge (nanoAcquity UPLC Trap Column; Waters), and then further separated on an analytical reversed phase C18 tip column (10 cm × ID 75 μm and 360 μm in diameter; Poly Micro Technologys). Mobile phase solvent A and B were prepared as 0.1% formic acid in water and 0.1% formic acid in acetonitrile, respectively. The separation condition was performed by eluting the peptides from the column with a linear gradient of 3%–40% B for 90 min, 40%–95% B for 2 min, 95% B for 10 min at a flow rate of 0.5 μL/min. The eluted peptides were ionized with spray voltage of 2.33 kV and introduced into the mass spectrometer. Mass spectrometric data was obtained using a data-dependent acquisition method (isolation width: 2 Da), in which one full MS survey scan (*m*/*z*: 200–1500) at a high resolution of 30,000 full at half maximum width was followed by MS/MS scan (*m*/*z*: 200–1500) of the six most intense multiply charged ions (2^+^ and 3^+^). Fragment ions of the each selected precursors were generated by collision-induced dissociation (CID) using helium gas with collision energy of 35% (or 3.5 eV). In addition, the dynamic exclusion duration of precursors was set to 120 s with an exclusion list size of 200.

### 2.7. Mass Spectrometric Data Analysis

LC-MS/MS raw data (.raw files) collected by using Xcalibur 2.0.7 SR1 (Thermo Electron, San Jose, CA, USA) were converted into peak list files (.dta) by using our in-house software within a Microsoft VBA environment. The resulting .dta files were applied to search against a UniProt rat protein database (containing 33,457 protein sequences; released on April, 2013; http://www.uniprot.org/) with an in-house TurboSequest search server (ver. 27, rev. 11; Thermo Electron, Waltham, MA, USA). The following search parameters were incorporated: peptide mass tolerance, 3.5 Da; fragment ion tolerance, 1 Da; enzyme set as trypsin; one missed cleavage allowed; peptide charge, 2^+^ and 3^+^; and oxidation on methionine (+16 Da), vinylpyridine alkylation on cysteine (+105.06 Da) allowed as variable modifications. TurboSequest results were filtered with the criteria similar to those of Qian *et al*. [23], and all accepted results had a DelCN (delta Cn) ≥ 0.1. A protein was identified when at least two unique peptides were matched with the Xcorr score for each peptide > 2.5. False-discovery rate (FDR, ≤1%) obtained from the search against the decoy database was used to estimate the protein identifications. Label-free quantitative analysis using MS spectra counting involved use of an in-house tool within the Microsoft VBA environment. MS spectral counts were normalized with the total identified spectra per biological sample and the proteins (containing at least two unique peptides) with statistically higher or lower peptide counts in PM-NAFLD rats (*t* test; *p*-value ≤ 0.05) were considered differentially expressed. All LC-MS/MS raw files in this study are accessible through the PeptideAtlas database (http://www.peptideatlas.org/) with the dataset identifier.

### 2.8. Ingenuity Pathway Analysis

The state-of-the-art pathway knowledge base Ingenuity^®^ Systems, Ingenuity Pathway Analysis (IPA) was used to infer global network functions of all differentially expressed proteins by PM-NAFLD. Accession numbers and expression fold change of the proteins were uploads into the IPA software for grouping the interaction networks and the biological functions of differential expression proteins. The significance (*p* value of overlap) was calculated by the Fisher’s exact test.

### 2.9. Statistical Analysis

All data are expressed as the mean ± SEM and analyzed by *t* test. *p* < 0.05 was considered statistically significant.

## 3. Results and Discussion

### 3.1. Plasma Estradiol Levels

**Figure 1:**
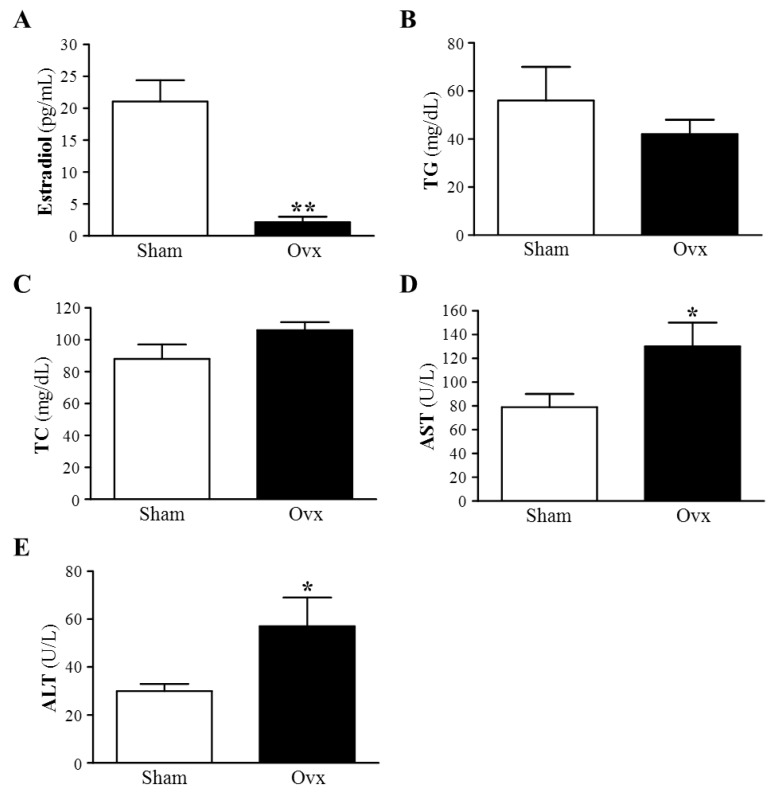
Effect of Ovx on plasma estradiol (**A**), TG (**B**), TC (**C**), AST (**D**) and ALT (**E**) levels at four months after ovariectomy. Mean ± SEM (*n* = 8). * *p* < 0.05 and ** *p* < 0.0001 *vs*. Sham, respectively.

The success of the ovariectomy was confirmed by examining plasma estradiol level. One week after ovariectomy, plasma estradiol level was significantly lower, by 85%, in the Ovx than Sham group (1.61 ± 0.72 *vs.* 10.49 ± 1.14 pg/mL, *p* < 0.0001). Until the end of the four-month study, the concentration of estradiol was significantly lower, by 90%, in Ovx than Sham group (*p* < 0.0001) (Figure 1A). In this study, plasma estradiol levels were markedly increased and served as evidence of the success of ovariectomy.

### 3.2. Plasma Lipid Profile: TG and TC

As compared with the Sham group, the Ovx group showed decreased plasma TG level, although not statistically significant (*p* = 0.343) (Figure 1B). The result was similar to previous observations by Liu *et al*. [24]. However, the Ovx group showed increased plasma TC level, although not significant (*p* = 0.0764), as compared with the Sham group (Figure 1C). Wattanapitayakul *et al*., also found no significant change in TC, TG, LDL, and HDL levels at weeks 0, 4, 8, and 12 after ovariectomy as compared with week 0 [25].

### 3.3. Plasma AST and ALT Activities

We further examined the liver function in the Ovx rat model by monitoring ALT and AST activities. The levels of plasma AST and ALT, markers of acute or chronic liver injury, were higher by 1.65-fold (*p* = 0.0391) and 2.0-fold (*p* = 0.0379), respectively, in the Ovx than Sham group, which supports the development of hepatocellular injury (Figure 1D,E). These results agree with earlier reports and indicate that serum indices of liver function by ovariectomy could be a secondary event after Ovx-induced lipid peroxidation of hepatocyte membranes with consequent increase in leakage of AST and ALT from liver tissue [26]. Wattanapitayakul *et al*., also found significant increases in serum ALT levels of Ovx rats at week 12 (*p* < 0.05 *vs*. week 0), with no change in AST levels [25]. Salim and Al-Rejaie showed that serum levels of hepatic enzymes, ALT and AST, were significantly increased in Ovx rats as compared with sham-operated animals [26].

### 3.4. Effect of Ovariectomy on Body Weight and Hepatic Mass

**Figure 2:**
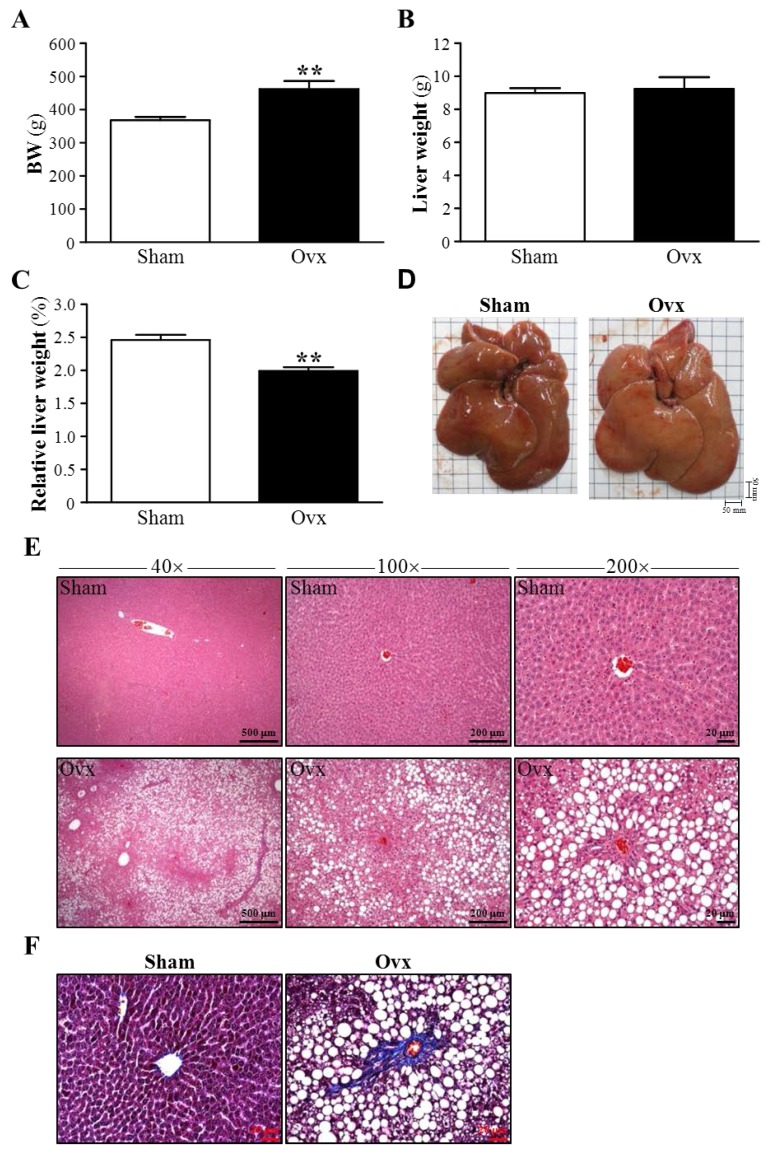
Effect of Ovx on liver gross and histopathology. (**A**) The body (**B**) liver weight and (**C**) liver mass/body weight ratio (relative liver weight, %) of Sham and Ovx rats at 4 months after ovariectomy. Mean ± SEM (*n* = 8). ** *p* < 0.005 versus Sham group; (**D**) Excised livers of Sham and Ovx rats; (**E**) Histological sections of the livers from Sham and Ovx rats with H&E staining. Specimens were photographed under a light microscope at 40×, 100×, and 200× magnification, and scale bars represent 500, 200, and 20 μm, respectively; (**F**) Histological sections of the livers from Sham and Ovx rats with Masson trichrome staining for collagenous scar tissue. Scale bars, 20 μm (magnification, 200×).

The success of ovariectomy was also confirmed by examining body weight. Body weight did not differ between Sham and Ovx groups (244 ± 2 g and 243 ± 5 g) at the beginning of study, but after four-month ovariectomy, body weight was higher for the Ovx group, by 1.26-fold, than the Sham group (*p* = 0.0029) (Figure 2A). These results agree with earlier reports and it is believed that the increased body weight is due to hormonal deficiency in menopausal women and is linked to increased weight of particularly epididymal fat [27,28,29]. The mean liver weights in the Sham and Ovx groups did not differ (8.99 ± 0.27 g and 9.24 ± 0.71 g, respectively, *p* = 0.7468) (Figure 2B). However, four months after ovariectomy, liver-to-body weight ratio was significantly decreased, by 19.36% (*p* = 0.0002), for the Ovx rats as compared with Sham rats (Figure 2C). Salim and Al-Rejaie also reported significantly decreased mean ratio of liver to body weight between Ovx and sham-operated rats (*p* < 0.01) [26]. As well, the liver of Sham rats was soft and pinkish brown, whereas the liver surface of Ovx rats was relatively harder and whiter than that for Sham rats (Figure 2D).

### 3.5. Effects of Ovariectomy on Hepatic Structure and Fibrosis

In conjunction with plasma levels of AST and ALT and the appearance of liver, the liver tissue of Ovx rats showed substantial histological alterations consistent with ovariectomy-induced hepatitis as compared with liver tissue from the Sham group. Notably, the Ovx rats showed massive changes in lipid deposition in liver sections as compared with Sham rats (Figure 2E). Ovariectomy also caused significant interstitial collagen deposition, as demonstrated by Masson’s trichrome staining of liver sections, which was not detected in the Sham group (Figure 2F).

### 3.6. Proteomic Analysis

**Figure 3:**
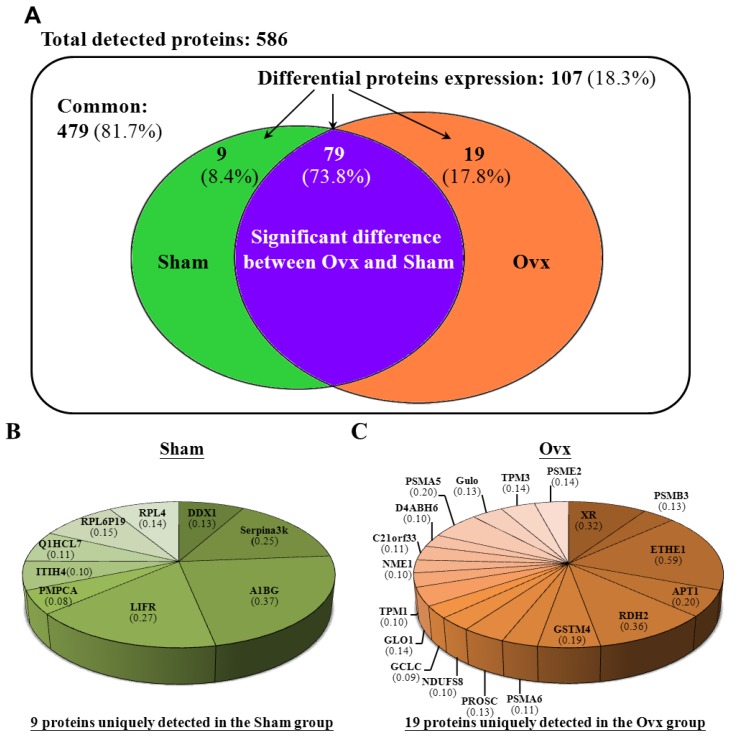

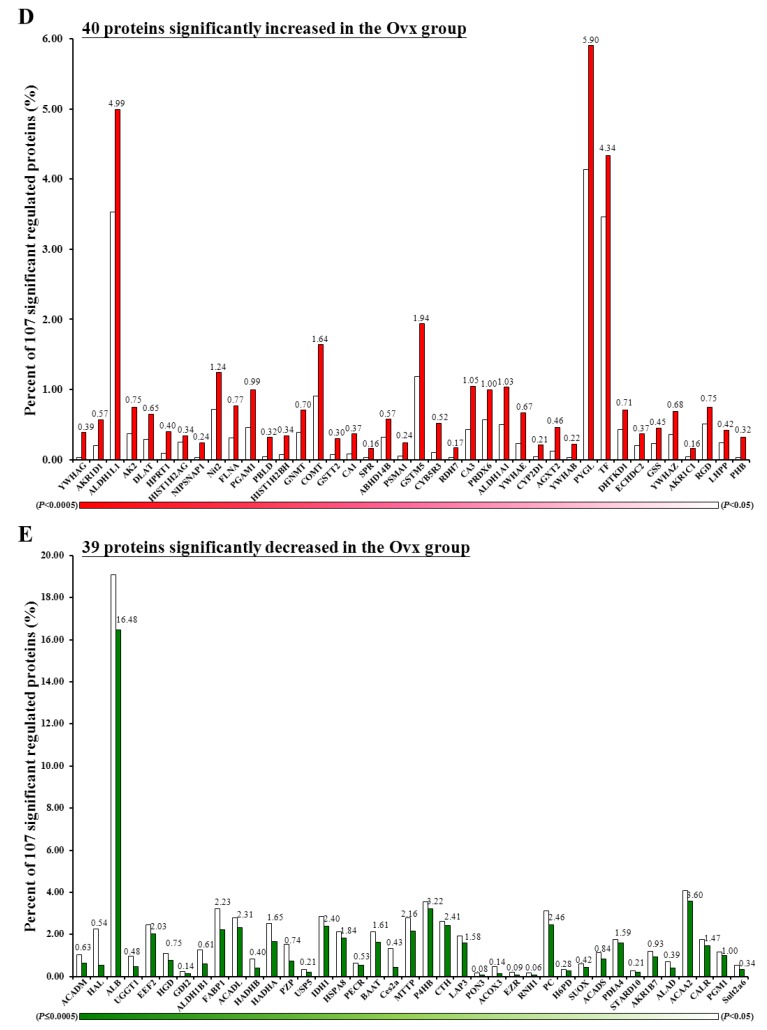
Venn diagrams comparing the common, only detected, or overlap of significantly regulated proteins in liver samples from Sham and Ovx rats. (**A**) Venn diagram of differentially expressed proteins; (**B**) Percentage distribution for the nine proteins detected only in the Sham group; (**C**) Percentage distribution for the 19 proteins detected only in the Ovx group; (**D**) Percentage distribution for the 40 proteins with significantly increased expression in the Ovx group compared to the Sham group; (**E**) Percentage distribution for the 39 proteins with significantly decreased expression in the Ovx group compared to the Sham group.

Hepatic proteins were separated by electrophoresis and digested by trypsin before being analyzed by tandem mass spectrometry. The spectra generated were analyzed by TurboSequest to identify the peptide sequences queried against the rat database in UniProt. The results were scored by using Xcorr. The proteins were considered hepatic proteins if more than two peptides from a single protein met the threshold Xcorr score (>2.5). The Venn diagram in Figure 3A summarizes the common, only detected, or overlapping proteins from the Sham and Ovx groups. There were 81.7% common and 18.3% significantly regulated proteins between the two groups. When the 81.7% un-changed proteins were removed and the other significantly regulated proteins were normalized to 100%, the Sham group showed 8.4% unique proteins and the Ovx group 17.8% unique proteins, with 73.8% overlapping regulated proteins between the groups (Figure 3A). In addition, the percent distribution for the 40 and 39 proteins was significantly increased and decreased, respectively, in the Ovx group compared to the Sham group (Figure 3D,E). More detailed peptide information for each proteome analysis can be found in Table 1 (Supplementary data), and related information.

**Figure 4:**
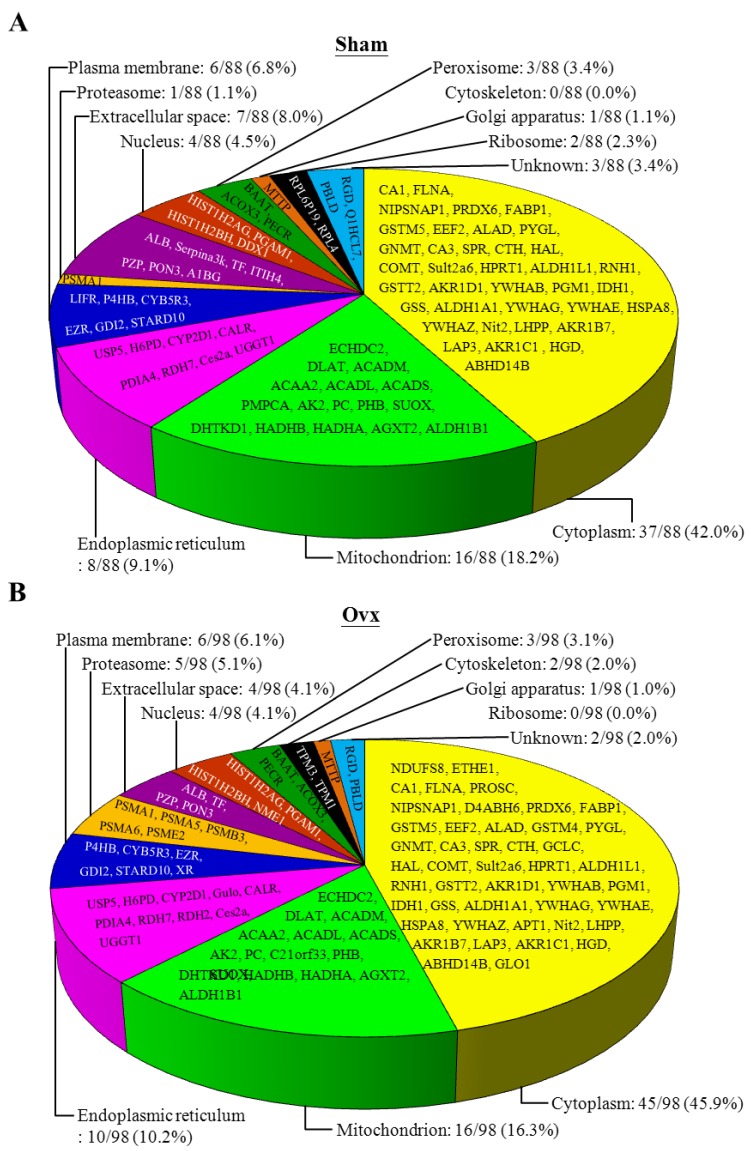
Classification of the differentially expressed proteins identified from Ovx and Sham groups. (**A**) Pie charts representing the distribution of the 88 differential proteins of the Sham group by their cellular location; (**B**) Pie charts representing the distribution of the 98 differential proteins of the Ovx group by their cellular location.

The 88 and 98 identified proteins with differential expression for the Sham and Ovx groups, respectively, were mostly in cytoplasm (37/88, Ovx group; 45/98, sham group), mitochondria (16/88, Ovx group; 16/98, sham group), and endoplasmic reticulum (8/88, Ovx group; 10/98, sham group) (Figure 4).

### 3.7. Ingenuity Pathways Analysis

**Figure 5:**
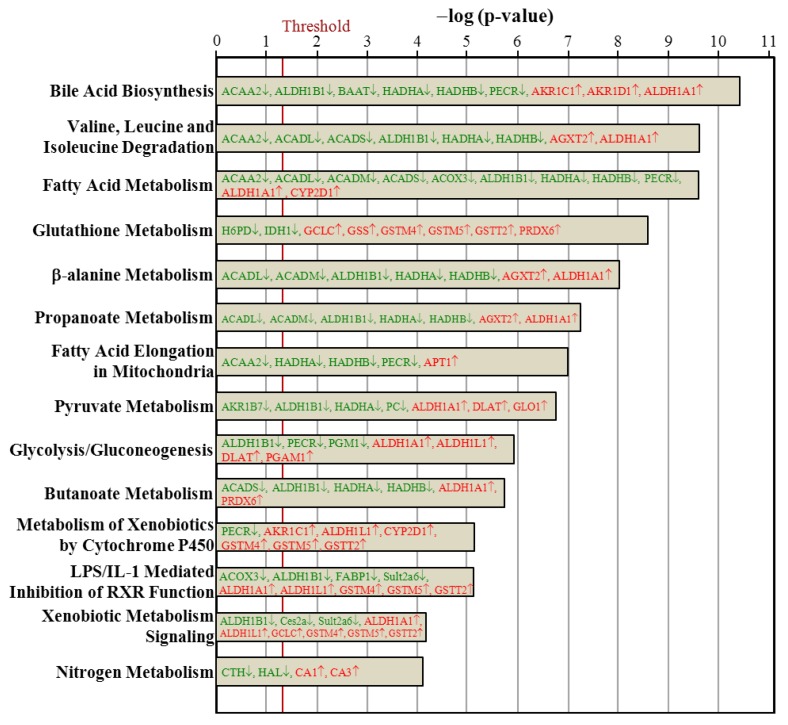
Comparison of canonical signaling pathways between the Ovx and Sham groups. The significantly enriched canonical pathways are displayed as a bar chart. Only the 14 most different pathways are shown, ranked by the significance in the Ovx group. The vertical line indicates a threshold of *p* < 0.05. Significantly enriched canonical pathways were determined by ingenuity systems pathways analyses and are displayed along the left *y*-axis. The *x*-axis displays the negative log of the *p*-value (blue bars), calculated by the right-tailed Fisher exact test.

**Figure 6:**
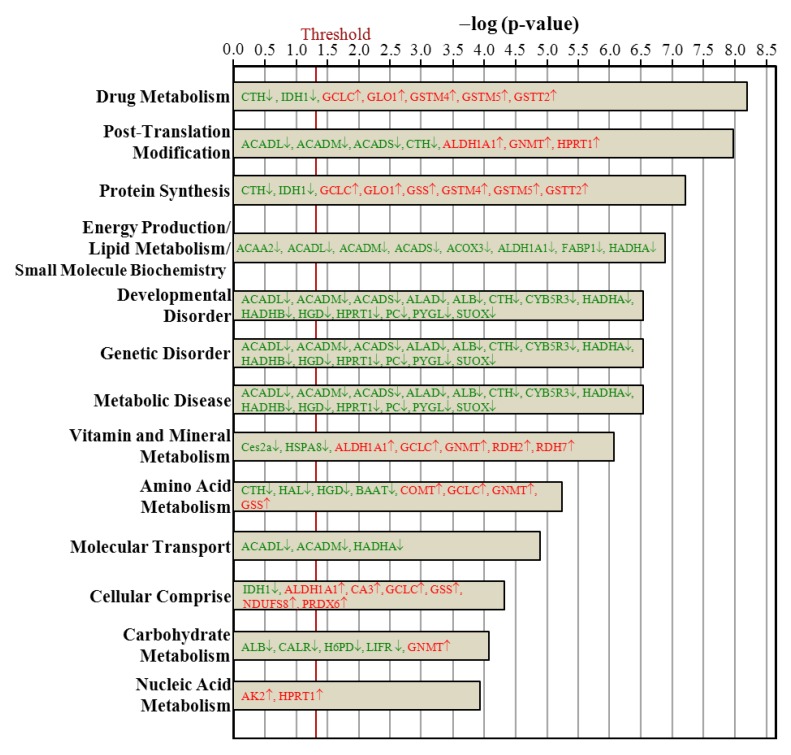
Comparison of biologic function proteins between the Ovx and Sham groups. Only the 15 most different biologic functions are shown, ranked by the significance in the Ovx group. The vertical line indicates a threshold of *p* < 0.05.

**Figure 7:**
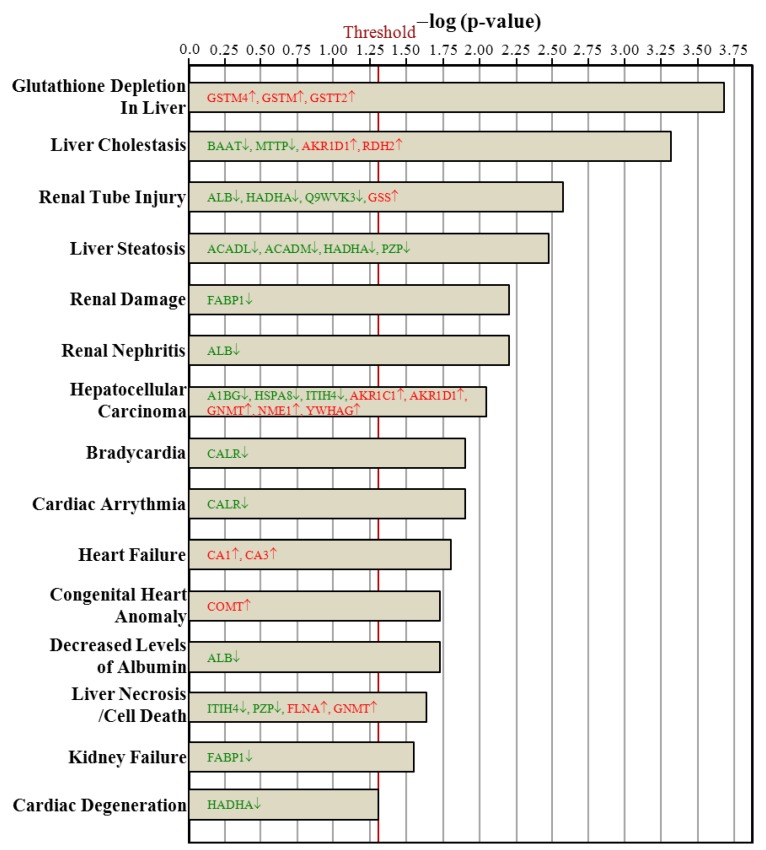
Comparison of toxic function proteins between the Ovx and Sham groups. Only the 15 most different toxic functions are shown, ranked by the significance in the Ovx group. The vertical line indicates a threshold of *p* < 0.05.

The hepatic proteins with changes in regulation due to ovariectomy could be classified into proteins implicated in canonical signaling pathways (Figure 5), biologic functions (Figure 6), and toxic functions (Figure 7). These three major pathways between the Ovx and Sham groups were generated by IPA with the threshold *p*-value < 0.05. The length of the bar indicates only that the differentially expressed proteins are related to this pathway but does not indicate the pathway as up- or downregulated.

The proteins regulated with ovariectomy were involved in bile acid biosynthesis; valine, leucine, and isoleucine degradation; fatty acid metabolism; glutathione metabolism; β-alanine metabolism; propanoate metabolism; fatty acid elongation in mitochondria; pyruvate metabolism; glycolysis/gluconeogenesis; butanoate metabolism; metabolism of xenobiotics by cytochrome P450; LPS/IL-1 mediated inhibition of retinoid X receptor (RXR) function; xenobiotic metabolism signaling; and nitrogen metabolism (Figure 5). The 13 biologic functions of the ovariectomy-regulated hepatic proteins implicated in PM-NAFLD were drug metabolism; post-translation modification; protein synthesis; energy production/lipid metabolism/small molecule biochemistry; developmental disorder; genetic disorder; metabolic disease; vitamin and mineral metabolism; amino acid metabolism; molecular transport; cellular comprise; carbohydrate metabolism; and nucleic acid metabolism (Figure 6). The toxic functions of ovariectomy-regulated hepatic proteins were glutathione depletion in liver; liver cholestasis; renal tube injury; liver steatosis; renal damage; renal nephritis; hepatocellular carcinoma; bradycardia; cardiac arrythmia; heart failure; congenital heart anomaly; decreased levels of albumin; liver necrosis/cell death; kidney failure; and cardiac degeneration (Figure 7).

Proteins in green letters were downregulated by ovariectomy and those in red were upregulated. Of these networks, two are involved in lipid metabolism of the rat liver phenotype. For proteins involved in fatty acid biosynthesis pathway, nine were downregulated and two were upregulated. The fatty acid biosynthesis pathway gave a *p*-value of 1.05 × 10^−7^, so this pathway was significantly changed by Ovx. The downregulated proteins included ACADM (1.82-fold, *p* = 0.0005), ALDH1B1 (2.28-fold, *p* = 0.0037), ACADL (1.32-fold, *p* = 0.0059), HADHB (2.27-fold, *p* = 0.0061), HADHA (1.69-fold, *p* = 0.0062), PECR (1.34-fold, *p* = 0.0146), ACOX3 (3.75-fold, *p* = 0.0215), ACADS (1.47-fold, *p* = 0.0312), ACAA2 (1.25-fold, *p* = 0.0413).

Living organisms are exposed to a large variety of fatty acids and their derivatives from exogenous sources or endogenous synthesis. The fatty acids and their CoA esters play multiple roles in cellular processes by serving as components in cellular lipids, carbon storage as TG, and substrates for α-, β-, and ω-oxidation. Fatty acid degradation in most organisms occurs primarily *via* the β-oxidation cycle, which in mammals takes place in both mitochondria and peroxisomes. However, mitochondrial β-oxidation is the main route for metabolism of fatty acids [30]. The first reaction within the mitochondrial matrix is acyl-CoA dehydrogenation catalyzed by acyl-CoA dehydrogenase (ACAD). Nine members were identified in the ACAD family, including the four that are involved in β-oxidation: short-, medium-, long-, and very-long-chain acyl-CoA dehydrogenase (ACADS, ACADM, ACADL, and ACADVL, respectively) [31]. Following dehydrogenation, enoyl-CoA hydratase catalyzes the formation of 3-hydroxyacyl-CoA, which is further followed by the formation of 3-ketoacyl-CoA. Finally, acetyl-CoA is generated through a thiolysis reaction. The last three steps of reactions are catalyzed by mitochondrial trifunctional protein (MTP), which is composed of an α-unit (HADHA) and a β-unit (HADHB). HADHA contains long-chain enoyl-CoA hydratase and 3-hydroxyacyl-CoA dehydrogenase activities, whereas HADHB contains the 3-ketoacyl-CoA thiolase activity [32]. In addition, ACAA2 catalyzes the last step in fatty acid oxidation to release acetyl CoA for Krebs cycle activity. According to Mannaerts *et al*., the contribution of peroxisomes to palmitate oxidation is only 5% of the overall fatty acid oxidation in isolated hepatocytes [33]. Thus, the metabolic fluxes due to fatty acid oxidation in the perfused livers appear to result predominantly from mitochondrial metabolism. In addition, the biologic role of peroxisomal β-oxidation system involves three enzymes: fatty acyl-CoA oxidase (ACOX), enoyl-CoA hydratase/3-hydroxyacyl-CoA dehydrogenase (HD), and thiolase. In the peroxisomal β-oxidation cycle, the first reaction is catalyzed by an ACOX, considered the main enzymatic step controlling the flux through the pathway [34].

Our proteomic analysis showed that the levels of ACADS, ACADM, ACADL, HADHA, HADHB, ACAA2, and ACOX3 were downregulated by ovariectomy. Downregulation of these proteins would limit the capacity of mitochondria and peroxisomes to oxidize fatty acids, thereby leading to hepatic steatosis. Mice deficient in ACADS show a fatty liver after dietary fat challenge [35]. ACADM is the most commonly inherited disorder of mitochondrial β-oxidation in humans. Mice deficient in ACADM also show a fatty liver [36]. MTP consists of four α and four β subunits that catalyze the final three steps of long-chain fatty acid β-oxidation. Newborn mice with the MTP α subunit (HADHA) null allele (*Mtpa*^−/−^) exhibit rapid development of hepatic steatosis after birth and significant necrosis of cardiac myocytes later [32]. Hepatic TC content and serum ALT activity were significantly higher in aging heterozygous Mtpa^+/−^ mice than Mtpa^+/+^ littermates [37]. During the first three to four months of age, the livers of ACOX^−/−^ mice exhibit severe microvesicular fatty metamorphosis of hepatocytes [38]. In this study, our finding of decreased expression of ACADS, ACADM, ACADL, HADHA, HADHB, and ACOX3 in the Ovx group may indicate attenuated oxidation of fatty acids and increased availability of fatty acids for synthesis.

## 4. Conclusions

NAFLD is common in postmenopausal women and associated with metabolic syndrome, which may be related to decreased fatty acid oxidation and increased lipogenesis in the liver, which causes excessive accumulation of hepatic fat and culminates in inflammation. Proteomics is a powerful tool to monitor the changes in protein profile after Ovx, and it may also provide potential biomarkers and further clinical applications for PM-NAFLD.

## Supplementary Materials

Supplementary materials can be accessed at: http://www.mdpi.com/2072-6643/7/10/5434/s1.
