# Supplementary material for: Proteomics Analysis to Identify and Characterize the Molecular Signatures of Hepatic Steatosis in Ovariectomized Rats as a Model of Postmenopausal Status
Source: Nutrients. 2015 Oct 22;7(10):8752–66. doi: 10.3390/nu7105434 (PMC4632454; doi:10.3390/nu7105434)
Supplement: Supplementary file 1 [file nutrients-07-05434-s001.docx]

**Supplementary Information**

**Table S1.** Differential liver protein expression between Sham and Ovx-treated rat ranked by *p*-value.

| **Description** | **Protein-ID** | **Abbrev.** | **Sham** | **Ovx** | ***p* value** | **Fold** |
| --- | --- | --- | --- | --- | --- | --- |
| 19 proteins uniquely detected in the Ovx group | | | | | | |
| L-xylulose reductase | Q920P0 | XR | 0 ± 0 | 3.81 ± 0.16 | <0.0001 |  |
| Proteasome subunit beta type-3 | P40112 | PSMB3 | 0 ± 0 | 1.56 ± 0.09 | <0.0001 |  |
| Ethylmalonic encephalopathy 1 | B0BNJ4 | ETHE1 | 0 ± 0 | 6.99 ± 0.46 | <0.0001 |  |
| Acyl-protein thioesterase 1 | P70470 | APT1 | 0 ± 0 | 2.33 ± 0.34 | 0.0005 |  |
| Retinol dehydrogenase 2 | P50170 | RDH2 | 0 ± 0 | 4.24 ± 0.80 | 0.0018 |  |
| Glutathione S-transferase Yb-3 | P08009 | GSTM4 | 0 ± 0 | 2.27 ± 0.51 | 0.0046 |  |
| Proteasome subunit alpha type-6 | P60901 | PSMA6 | 0 ± 0 | 1.32 ± 0.44 | 0.0242 |  |
| Proline synthetase co-transcribed (predicted) | D3ZCA0 | PROSC | 0 ± 0 | 1.58 ± 0.53 | 0.0250 |  |
| NADH dehydrogenase (Ubiquinone) Fe-S protein 8 (predicted), isoform CRA_a | B0BNE6 | NDUFS8 | 0 ± 0 | 1.22 ± 0.41 | 0.0254 |  |
| Glutamate--cysteine ligase catalytic subunit | P19468 | GCLC | 0 ± 0 | 1.10 ± 0.37 | 0.0263 |  |
| Lactoylglutathione lyase | Q6P7Q4 | GLO1 | 0 ± 0 | 1.64 ± 0.56 | 0.0273 |  |
| Tropomyosin alpha-1 chain | P04692 | TPM1 | 0 ± 0 | 1.24 ± 0.43 | 0.0295 |  |
| Nucleoside diphosphate kinase A | Q05982 | NME1 | 0 ± 0 | 1.15 ± 0.41 | 0.0299 |  |
| ES1 protein homolog, mitochondrial | P56571 | C21 or f33 | 0 ± 0 | 1.32 ± 0.47 | 0.0307 |  |
| Uncharacterized protein | D4ABH6 |  | 0 ± 0 | 1.14 ± 0.41 | 0.0308 |  |
| Proteasome subunit alpha type-5 | P34064 | PSMA5 | 0 ± 0 | 2.41 ± 0.88 | 0.0339 |  |
| L-gulonolactone oxidase | P10867 | Gulo | 0 ± 0 | 1.53 ± 0.57 | 0.0369 |  |
| Tropomyosin alpha-3 chain | Q63610 | TPM3 | 0 ± 0 | 1.67 ± 0.63 | 0.0390 |  |
| Proteasome activator complex subunit 2 | Q63798 | PSME2 | 0 ± 0 | 1.66 ± 0.67 | 0.0472 |  |

**Table S1.** *Cont.*

| **Description** | **Protein-ID** | **Abbrev.** | **Sham** | **Ovx** | ***p* value** | **Fold** |
| --- | --- | --- | --- | --- | --- | --- |
| 40 proteins significantly increased in the Ovx group | | | | | | |
| 14-3-3 protein gamma | P61983 | YWHAG | 0.37 ± 0.37 | 4.60 ± 0.46 | 0.0004 | +12.57 |
| 3-oxo-5-beta-steroid 4-dehydrogenase | P31210 | AKR1D1 | 2.69 ± 0.47 | 6.81 ± 0.66 | 0.0023 | +2.53 |
| 10-formyltetrahydrofolate dehydrogenase | P28037 | ALDH1L1 | 46.31 ± 0.81 | 59.54 ± 2.56 | 0.0026 | +1.29 |
| Adenylate kinase 2, mitochondrial | P29410 | AK2 | 4.89 ± 0.45 | 8.96 ± 0.70 | 0.0027 | +1.83 |
| Dihydrolipoyllysine-residue acetyltransferase component of pyruvate dehydrogenase complex, mitochondrial | P08461 | DLAT | 3.78 ± 0.63 | 7.71 ± 0.52 | 0.0030 | +2.04 |
| Hypoxanthine-guanine phosphoribosyltransferase | P27605 | HPRT1 | 1.23 ± 0.74 | 4.81 ± 0.17 | 0.0033 | +3.91 |
| Histone H2A type 1-C | P0C169 | HIST1H2AG | 3.30 ± 0.13 | 4.05 ± 0.11 | 0.0047 | +1.23 |
| Uncharacterized protein | D4A867 | NIPSNAP1 | 0.37 ± 0.37 | 2.89 ± 0.45 | 0.0051 | +7.76 |
| Omega-amidase NIT2 | Q497B0 | Nit2 | 9.41 ± 0.82 | 14.78 ± 0.97 | 0.0055 | +1.57 |
| Filamin alpha | C0JPT7 | FLNA | 4.03 ± 0.36 | 9.13 ± 1.16 | 0.0057 | +2.27 |
| Phosphoglycerate mutase 1 | P25113 | PGAM1 | 5.99 ± 0.94 | 11.85 ± 1.04 | 0.0059 | +1.98 |
| Phenazine biosynthesis-like domain-containing protein | Q68G31 | PBLD | 0.50 ± 0.50 | 3.78 ± 0.62 | 0.0063 | +7.61 |
| Histone H2B type 1 | Q00715 | HIST1H2BH | 0.97 ± 0.56 | 4.04 ± 0.50 | 0.0064 | +4.16 |
| Glycine N-methyltransferase | P13255 | GNMT | 5.14 ± 0.61 | 8.40 ± 0.53 | 0.0069 | +1.63 |
| Catechol O-methyltransferase | P22734 | COMT | 11.86 ± 1.73 | 19.53 ± 0.90 | 0.0077 | +1.65 |
| Glutathione S-transferase theta-2 | P30713 | GSTT2 | 0.97 ± 0.6 | 3.56 ± 0.32 | 0.0086 | +3.66 |
| Carbonic anhydrase 1 | B0BNN3 | CA1 | 1.10 ± 0.7 | 4.36 ± 0.54 | 0.0102 | +3.97 |
| Sepiapterin reductase | P18297 | SPR | 0.36 ± 0.36 | 1.91 ± 0.21 | 0.0104 | +5.24 |
| Abhydrolase domain-containing protein 14B | Q6DGG1 | ABHD14B | 4.16 ± 0.44 | 6.85 ± 0.60 | 0.0109 | +1.65 |
| Proteasome subunit alpha type-1 | P18420 | PSMA1 | 0.74 ± 0.43 | 2.92 ± 0.43 | 0.0113 | +3.96 |
| Glutathione S-transferase Mu 1 | P04905 | GSTM5 | 15.53 ± 1.17 | 23.08 ± 1.76 | 0.0117 | +1.49 |
| NADH-cytochrome b5 reductase 3 | P20070 | CYB5R3 | 1.35 ± 0.79 | 6.21 ± 1.17 | 0.0138 | +4.59 |

**Table S1.** *Cont.*

| **Description** | **Protein-ID** | **Abbrev.** | **Sham** | **Ovx** | ***p* value** | **Fold** |
| --- | --- | --- | --- | --- | --- | --- |
| Retinol dehydrogenase 3 | P50169 | RDH7 | 0.37 ± 0.37 | 2.03 ± 0.32 | 0.0149 | +5.45 |
| Carbonic anhydrase 3 | P14141 | CA3 | 5.62 ± 0.75 | 12.49 ± 1.98 | 0.0176 | +2.22 |
| Peroxiredoxin-6 | O35244 | PRDX6 | 7.46 ± 1.08 | 11.88 ± 0.83 | 0.0178 | +1.59 |
| Retinal dehydrogenase 1 | P51647 | ALDH1A1 | 6.48 ± 0.68 | 12.32 ± 1.68 | 0.0181 | +1.90 |
| 14-3-3 protein epsilon | P62260 | YWHAE | 3.07 ± 1.38 | 8.01 ± 0.68 | 0.0182 | +2.61 |
| Cytochrome P450 2D1 | P10633 | CYP2D1 | 0.49 ± 0.49 | 2.46 ± 0.38 | 0.0188 | +5.05 |
| Alanine--glyoxylate aminotransferase 2, mitochondrial | Q64565 | AGXT2 | 1.58 ± 0.96 | 5.43 ± 0.78 | 0.0203 | +3.45 |
| 14-3-3 protein beta/alpha | P35213 | YWHAB | 0.37 ± 0.37 | 2.67 ± 0.64 | 0.0210 | +7.18 |
| Glycogen phosphorylase, liver form | P09811 | PYGL | 54.16 ± 3.54 | 70.36 ± 3.85 | 0.0211 | +1.30 |
| Serotransferrin | P12346 | TF | 45.30 ± 1.12 | 51.73 ± 1.88 | 0.0261 | +1.14 |
| Dehydrogenase E1 and transketolase domain containing 1, mitochondrial | Q4KLP0 | DHTKD1 | 5.61 ± 0.74 | 8.44 ± 0.66 | 0.0288 | +1.50 |
| Enoyl Coenzyme A hydratase domain containing 2 (predicted), isoform CRA_a | D3ZIL6 | ECHDC2 | 2.57 ± 0.23 | 4.38 ± 0.60 | 0.0303 | +1.71 |
| Glutathione synthetase | P46413 | GSS | 3.05 ± 0.3 | 5.34 ± 0.76 | 0.0309 | +1.75 |
| 14-3-3 protein zeta/delta | P63102 | YWHAZ | 4.66 ± 1.05 | 8.16 ± 0.70 | 0.0328 | +1.75 |
| Aldo-keto reductase family 1 member C21 | Q6AYQ2 | AKR1C1 | 0.49 ± 0.49 | 1.90 ± 0.20 | 0.0360 | +3.92 |
| RGD1564894 protein | B1H250 | RGD | 6.72 ± 0.43 | 8.92 ± 0.75 | 0.0442 | +1.33 |
| Phospholysine phosphohistidine inorganic pyrophosphate phosphatase | Q5I0D5 | LHPP | 3.17 ± 0.57 | 5.02 ± 0.48 | 0.0475 | +1.58 |
| Prohibitin | P67779 | PHB | 0.36 ± 0.36 | 3.87 ± 1.38 | 0.0493 | +10.63 |

**Table S1.** *Cont.*

| **Description** | **Protein-ID** | **Abbrev.** | **Sham** | **Ovx** | ***p* value** | **Fold** |
| --- | --- | --- | --- | --- | --- | --- |
| 9 proteins non-detected in the Ovx group but uniquely detected in the Sham group | | | | | | |
| ATP-dependent RNA helicase DDX1 | Q641Y8 | DDX1 | 1.71 ± 0.13 | 0 ± 0 | <0.0001 |  |
| Serine protease inhibitor A3K | P05545 | Serpina3k | 3.30 ± 0.24 | 0 ± 0 | <0.0001 |  |
| Alpha-1B-glycoprotein | Q9EPH1 | A1BG | 4.89 ± 0.69 | 0 ± 0 | 0.0004 |  |
| Leukemia inhibitory factor receptor | O70535 | LIFR | 3.55 ± 0.81 | 0 ± 0 | 0.0047 |  |
| Mitochondrial-processing peptidase subunit alpha | P20069 | PMPCA | 1.09 ± 0.36 | 0 ± 0 | 0.0240 |  |
| Inter alpha-trypsin inhibitor, heavy chain 4 | Q5EBC0 | ITIH4 | 1.34 ± 0.46 | 0 ± 0 | 0.0267 |  |
| UPF0465 protein C5orf33 homolog | Q1HCL7 |  | 1.47 ± 0.53 | 0 ± 0 | 0.0328 |  |
| 60S ribosomal protein L6 | P21533 | RPL6P19 | 1.96 ± 0.77 | 0 ± 0 | 0.0446 |  |
| 60S ribosomal protein L4 | P50878 | RPL4 | 1.83 ± 0.72 | 0 ± 0 | 0.0448 |  |
| 39 proteins significantly decreased in the Ovx group | | | | | | |
| Medium-chain specific acyl-CoA dehydrogenase, mitochondrial | P08503 | ACADM | 13.68 ± 0.63 | 7.52 ± 0.62 | 0.0005 | −1.82 |
| Histidine ammonia-lyase | P21213 | HAL | 29.43 ± 3.31 | 6.49 ± 0.81 | 0.0005 | −4.54 |
| Serum albumin | P02770 | ALB | 250.25 ± 7.92 | 196.50 ± 3.67 | 0.0008 | −1.27 |
| UDP-glucose:glycoprotein glucosyltransferase 1 | Q9JLA3 | UGGT1 | 12.82 ± 0.7 | 5.77 ± 0.97 | 0.0011 | −2.22 |
| Elongation factor 2 | P05197 | EEF2 | 32.24 ± 1.12 | 24.18 ± 0.86 | 0.0013 | −1.33 |
| Homogentisate 1, 2-dioxygenase | Q6AYR0 | HGD | 14.54 ± 0.91 | 8.96 ± 0.61 | 0.0022 | −1.62 |
| Rab GDP dissociation inhibitor beta | P50399 | GDI2 | 3.30 ± 0.22 | 1.70 ± 0.26 | 0.0033 | −1.94 |
| Aldehyde dehydrogenase X, mitochondrial | Q66HF8 | ALDH1B1 | 16.64 ± 1.79 | 7.30 ± 0.97 | 0.0037 | −2.28 |
| Fatty acid-binding protein, liver | P02692 | FABP1 | 42.13 ± 2.66 | 26.56 ± 2.17 | 0.0040 | −1.59 |
| Long-chain specific acyl-CoA dehydrogenase, mitochondrial | P15650 | ACADL | 36.39 ± 2.1 | 27.58 ± 0.3 | 0.0059 | −1.32 |
| Trifunctional enzyme subunit beta, mitochondrial | Q60587 | HADHB | 10.76 ± 0.97 | 4.73 ± 1.09 | 0.0061 | −2.27 |

**Table S1.** *Cont.*

| **Description** | **Protein-ID** | **Abbrev.** | **Sham** | **Ovx** | ***p* value** | **Fold** |
| --- | --- | --- | --- | --- | --- | --- |
| Trifunctional enzyme subunit alpha, mitochondrial | Q64428 | HADHA | 33.13 ± 2.29 | 19.63 ± 2.34 | 0.0062 | −1.69 |
| Alpha-1-macroglobulin | Q63041 | PZP | 19.90 ± 1.85 | 8.84 ± 2.19 | 0.0084 | −2.25 |
| Ubiquitin specific protease 5 (Isopeptidase T) (predicted) | D3ZVQ0 | USP5 | 4.64 ± 0.5 | 2.49 ± 0.29 | 0.0097 | −1.86 |
| Isocitrate dehydrogenase [NADP] cytoplasmic | P41562 | IDH1 | 37.50 ± 1.41 | 28.67 ± 2.1 | 0.0130 | −1.31 |
| Heat shock cognate 71 kDa protein | P63018 | HSPA8 | 27.97 ± 1.1 | 21.96 ± 1.37 | 0.0142 | −1.27 |
| Peroxisomal trans-2-enoyl-CoA reductase | Q9WVK3 | PECR | 8.43 ± 0.63 | 6.28 ± 0.05 | 0.0146 | −1.34 |
| Bile acid-CoA:amino acid N-acyltransferase | Q63276 | BAAT | 27.84 ± 2.21 | 19.24 ± 1.26 | 0.0149 | −1.45 |
| Carboxylesterase isoenzyme | Q8K3R0 | Ces2a | 17.58 ± 3.17 | 5.14 ± 1.9 | 0.0152 | −3.42 |
| Microsomal triglyceride transfer protein | D4A1W8 | MTTP | 36.39 ± 2.8 | 25.72 ± 1.69 | 0.0173 | −1.41 |
| Protein disulfide-isomerase | P04785 | P4HB | 46.56 ± 1.58 | 38.42 ± 1.96 | 0.0179 | −1.21 |
| Cystathionine gamma-lyase | P18757 | CTH | 34.18 ± 1.38 | 28.76 ± 0.96 | 0.0180 | −1.19 |
| Cytosol aminopeptidase | Q68FS4 | LAP3 | 25.28 ± 0.95 | 18.85 ± 1.76 | 0.0184 | −1.34 |
| Serum paraoxonase/lactonase 3 | Q68FP2 | PON3 | 2.08 ± 0.12 | 0.99 ± 0.33 | 0.0212 | −2.10 |
| Peroxisomal acyl-coenzyme A oxidase 3 | Q63448 | ACOX3 | 6.10 ± 1.01 | 1.63 ± 1.03 | 0.0215 | −3.75 |
| Ezrin | P31977 | EZR | 2.56 ± 0.36 | 1.03 ± 0.34 | 0.0221 | −2.48 |
| Ribonuclease inhibitor | P29315 | RNH1 | 2.19 ± 0.31 | 0.69 ± 0.40 | 0.0242 | −3.18 |
| Pyruvate carboxylase, mitochondrial | P52873 | PC | 40.77 ± 2.58 | 29.27 ± 3.01 | 0.0273 | −1.39 |
| Hexose-6-phosphate dehydrogenase (Glucose 1-dehydrogenase) (predicted), isoform CRA_b | D4A7D7 | H6PD | 4.28 ± 0.31 | 3.36 ± 0.07 | 0.0280 | −1.27 |
| Sulfite oxidase, mitochondrial | Q07116 | SUOX | 7.95 ± 0.80 | 5.02 ± 0.64 | 0.0295 | −1.58 |
| Short-chain specific acyl-CoA dehydrogenase, mitochondrial | P15651 | ACADS | 14.64 ± 1.10 | 9.97 ± 1.26 | 0.0312 | −1.47 |
| Protein disulfide-isomerase A4 | P38659 | PDIA4 | 23.09 ± 1.24 | 19.01 ± 0.88 | 0.0362 | −1.21 |
| START domain containing 10, isoform CRA_b | Q5BJN1 | STARD10 | 3.67 ± 0.33 | 2.44 ± 0.32 | 0.0373 | −1.50 |

**Table S1.** *Cont.*

| **Description** | **Protein-ID** | **Abbrev.** | **Sham** | **Ovx** | ***p* value** | **Fold** |
| --- | --- | --- | --- | --- | --- | --- |
| Aldo-keto reductase family 1, member B7 | Q5RJP0 | AKR1B7 | 15.52 ± 1.58 | 11.03 ± 0.59 | 0.0373 | −1.41 |
| Delta-aminolevulinic acid dehydratase | P06214 | ALAD | 9.41 ± 1.65 | 4.70 ± 0.73 | 0.0395 | −2.01 |
| 3-ketoacyl-CoA thiolase, mitochondrial | P13437 | ACAA2 | 53.58 ± 2.74 | 42.87 ± 3.1 | 0.0413 | −1.25 |
| Calreticulin | P18418 | CALR | 23.09 ± 1.95 | 17.56 ± 0.90 | 0.0423 | −1.31 |
| Phosphoglucomutase-1 | P38652 | PGM1 | 15.25 ± 1.07 | 11.89 ± 0.78 | 0.0434 | −1.28 |
| Alcohol sulfotransferase A | P22789 | Sult2a6 | 7.20 ± 0.99 | 4.04 ± 0.81 | 0.0484 | −1.78 |

^1^ Table 1 is a list of uniquely detected, significantly increased, non-detected, and significantly decreased liver proteome at 4-month after ovariectomy, as ranked by
*p*-value. *p* values were analyzed by paired Student’s *t*-test. ^2^ Data were expressed by Mean ± SEM from 4 independent samples. ^3^ Protein description, database name, and ID relate to the Swiss-Prot database (57.1) entry returned using the MASCOT search engine. A MOWSE score greater than 51 denotes a confident (*p* < 0.05) protein identification. Sham and Ovx expression ratios were the Mean ± SEM of peptides quantified in each sample expressed relative to the pooled internal standard. Fold difference relative to Sham values was reported and *p-*values were determined from log-transformed data using Student’s independent *t*-tests. The complete list of proteins identified by iTRAQ and the Mean ± SEM number of MS/MS ions analyzed and the Mean ± SEM peptides that met the inclusion criteria for iTRAQ analysis (including amino acid sequences identified) were reported.

© 2015 by the authors; licensee MDPI, Basel, Switzerland. This article is an open access article distributed under the terms and conditions of the Creative Commons by Attribution (CC-BY) license (http://creativecommons.org/licenses/by/4.0/).
